# Physically Cross-Linked Gels of PVA with Natural Polymers as Matrices for Manuka Honey Release in Wound-Care Applications

**DOI:** 10.3390/ma12040559

**Published:** 2019-02-13

**Authors:** Antonia Monica Neres Santos, Ana Paula Duarte Moreira, Carlos W. Piler Carvalho, Rosa Luchese, Edlene Ribeiro, Garrett B. McGuinness, Marisa Fernandes Mendes, Renata Nunes Oliveira

**Affiliations:** 1 Postgraduate Program of Chemical Engineering/DEQ, UFRRJ, Seropédica-RJ 23890-000, Brazil; antoniamonicaa@hotmail.com (A.M.N.S.); marisamendes@globo.com (M.F.M.); 2 Materials and Metallurgy Engineering Program/COPPE, UFRJ, Seropédica-RJ 23890-000, Brazil; duarteap@gmail.com; 3 Brazilian Agricultural Research Corporation/Embrapa Food Technology, Brasília 70770-901, Brazil; carlos.piler@embrapa.br; 4 Department of food engineering, UFRRJ, Seropédica-RJ 23890-000, Brazil; rhluche@gmail.com (R.L.); edleneribeiro@gmail.com (E.R.); 5 Centre for Medical Engineering Research, School of Mechanical and Manufacturing Engineering, Dublin City University, Dublin 9, Ireland; garrett.mcguinness@dcu.ie

**Keywords:** PVA blends, manuka honey, natural polymers, burn care

## Abstract

Manuka honey is a well-known natural material from New Zealand, considered to have properties beneficial for burn treatment. Gels created from polyvinyl alcohol (PVA) blended with natural polymers are potential burn-care dressings, combining biocompatibility with high fluid uptake. Controlled release of manuka honey from such materials is a possible strategy for improving burn healing. This work aimed to produce polyvinyl alcohol (PVA), PVA–sodium carboxymethylcellulose (PVA-CMC), PVA–gelatin (PVA-G), and PVA–starch (PVA-S) cryogels infused with honey and to characterize these materials physicochemically, morphologically, and thermally, followed by in vitro analysis of swelling capacity, degradation/weight loss, honey delivery kinetics, and possible activity against *Staphylococcus aureus*. The addition of honey to PVA led to many PVA crystals with defects, while PVA–starch–honey and PVA–sodium carboxymethylcellulose–honey (PVA-CMC-H) formed amorphous gels. PVA-CMC presented the highest swelling degree of all. PVA-CMC-H and PVA–gelatin–honey presented the highest swelling capacities of the honey-laden samples. Weight loss/degradation was significantly higher for samples containing honey. Layers submitted to more freeze–thawing cycles were less porous in SEM images. With the honey concentration used, samples did not inhibit *S. aureus*, but pure manuka honey was bactericidal and dilutions superior to 25% honey were bacteriostatic, indicating the need for higher concentrations to be more effective.

## 1. Introduction

According to the American Burn Association, between 2008 and 2017, there were more than 212,000 burn-related admissions in United States of America (USA) hospitals, 23% of whom were between one and 15 years of age. Most of their burn injuries occurred at home, with scald injuries being the most common among children under five years of age. Burn injuries incur high care costs, where the average charges for surviving and non-surviving patients were $269,523 and $361,342, respectively [1,2]. Partial thickness skin burns (characterized by swelling, blistering, and redness) can cause physical pain and scarring, as well as potentially associated issues such as anxiety and depression. Wounds are typically treated by cleansing the site and applying wound dressings [3]. Among the characteristics of an ideal burn dressing should be a moist environment that stimulates healing, the capacity to absorb the wound exudate, inhibition of bacterial growth and infection, non-adherence to the injured skin, and transparency or translucidity to facilitate monitoring of the healing process [4,5]. Burn infections (such as *Staphylococcus aureus* growth from the human skin flora) can be considered an important variable that impedes the healing and potentially leads to patients’ death [6]. To prevent infection, several dressings containing natural or synthetic antimicrobials were developed and studied. For example, 2-acrylamido-2-methylpropane sulfonic acid sodium salt hydrogels containing nano-silver showed no cytotoxicity, delivered at least 70% of the loaded silver to the burn site in 72 h, and exhibited antimicrobial activity against both *S. aureus* and *Pseudomonas aeruginosa* [7]. In another study, silver-impregnated dressings and silver nanocrystals were compared, and the nanocrystalline silver was considered the more effective antibacterial system that also stimulated healing [8]. Finally, a systematic review revealed an indication that honey dressings promote better burn healing than silver sulfadiazine ones [9].

Honey is a natural material produced by bees and it was used in wound treatment for centuries. The development of antibiotics in the 20th century discouraged the use of natural materials for wound treatment; however, antibiotic-resistant microorganisms such as methicillin-resistant *S. aureus* turned the wound-care research field’s attention back to natural products [10]. The activity of honey is usually attributed to H_2_O_2_ production, derived from the glucose oxidase enzyme [11]. Among the available types of honey, manuka honey is a medical-grade monofloral (from *Leptospermum scoparium* tree pollen) honey from New Zealand and Australia. Its activity is related to the 1,2-dicarbonyl compound methylglyoxal (MGO), to its non-peroxidase activity, where the concentration of MGO in the honey determines the unique manuka factor (UMF). Antibacterial manuka honeys are the ones with concentrations superior to 0.15 mg/g MGO [12,13,14]. In addition, its dark color could be related to high proportions of phenolic compounds, which present antimicrobial and anti-oxidant properties [15]. Honey, when applied to burns, promotes a moist environment which stimulates healing. It guarantees re-epithelization and non-adherence of the applied dressing to the wound site, protecting the newly formed tissue [16]. 

A moist environment favors burn healing, leading burn-care research toward hydrogel dressings. Alginate hydrogels loaded with thymol extracted from Iranian honeys presented bactericide effects (the gel was tested against *S. aureus, Klebsiella pneumoniae, Acinetobacter baumannii,* and *P. aeruginosa* microorganisms) and stimulated burn healing in rats [17]. Chitosan gels loaded with Egyptian honey (75%) showed high antimicrobial activity (the organisms tested were *Pseudomonas aeruginosa, Staphylococcus aureus, Klebsiella pneumonia*, and *Streptococcus pyogenes*) and a high rate of burn healing in mice [18]. When added to chitosan hydrogels, the effect of manuka honey is dose-dependent, where lower honey concentrations lead to increased swelling of the gel [19]. Manuka honey tulle dressings keep wounds moist, which helps debridement of necrotic tissue of chronic wounds infected with methicillin-resistant *S. aureus* (MRSA) [20]. Chitosan–gelatin–manuka honey extract hydrogels absorb exudate and present antimicrobial and healing effects [21]. Dextran–nanosoy–glycerol–chitosan nanocomposite membranes containing *Aloe vera* and manuka honey presented an initial burst release followed by a controlled release, and presented activity against *Staphylococcus aureus* and *Escherichia coli* [22]. Silk fibroin–manuka honey electrospun mats presented biocompatibility, bactericide action against *E. coli, S. aureus, P. aeruginosa*, MRSA (manuka honey dose-dependent property), and improved wound healing in mice [23]. As a proposed dressing for wounds with moderate amounts of exudate, polyvinyl alcohol (PVA)–manuka honey hydrogels cross-linked with borax presented antimicrobial activity, sustained honey release, and high cell viability and proliferation [24].

As discussed above, manuka honey was incorporated within several hydrogel formulations as research platforms. Hydrogels are cross-linked networks of hydrophilic polymers that swell in aqueous media, presenting many similar characteristics to human soft tissue [25]. Burns treated with hydrogel dressings presented faster healing than those treated with alternative options (paraffin gauze or silver sulfadiazine or paraffin gauze containing antibiotics) [26]. In addition, due to their high water/fluid uptake, hydrogels not only keep a moist environment that facilitates the migration of cells, but they also present a cooling effect which helps relieve pain [5]. Several hydrogels were prepared specifically for burn healing applications. Polyvinylpyrrolidone iodine hydrogels healed minor burns faster than silver sulfadiazine cream, but the hydrogel anti-inflammatory properties were not accessed [27]. Polyvinyl alcohol (7.5%)–chitosan (0.75%) freeze–thawed gels loaded with silver sulfadiazine presented sufficient gel strength and fluid uptake for potential burn-care applications [28]. Polyvinyl alcohol, abbreviated as PVA, is a well-known biocompatible synthetic polymer used as a raw material for wound-care hydrogel production [29]. Natural polymers were also considered for hydrogel dressings since they are biocompatible and, usually, biodegradable. Examples include cellulose, chitosan, and starch [30]. Sodium carboxymethyl cellulose (2%)–sodium alginate (3%)–chitosan (1%) non-adhesive gels promoted second-degree burn healing in rats [5]. Hydrogels containing gelatin, hyaluronic acid, chondroitin sulfate, asiatic acid, zinc oxide, and copper oxide nanoparticles also helped second-degree burns in Wistar rats to heal [31]. 

As mentioned, gels fabricated from PVA, or blends of PVA with other constituents, and containing manuka honey were previously studied. PVA gels can be formed through chemical, irradiation [32], or physical cross-linking. Physical cross-linking is usually preferred since the potential toxicity of chemical cross-linking additives are avoided [29]. 

In one study, gamma-irradiated gels were formed from PVA blended with gelatin and chitosan, combined with polycaprolactone (PCL) microspheres containing basic fibroblast growth factor (bFGF). These gels presented burst and sustained bFGF release, no cytotoxicity, and potential for fibroblast proliferation [33]. Gelatin is a natural protein derived from the hydrolysis of collagen, presenting solubility in aqueous fluids, and it facilitates cell adhesion and proliferation. When combined with PVA physically, gelatin can present some phase separation [34], but 0.01% gelatin was enough to increase the PVA–tyramine hydrogel biofunctionality and cell adhesion [35]. 

Starch is a hydrophilic natural polysaccharide and does not form stable hydrogels by itself. Starch was mixed with PVA and hydrogels were chemically formed (by adding glutaraldehyde) and loaded with turmeric. The resulting gels were bactericidal (active against Gram-positive and Gram-negative organisms) [36]. PVA–starch hydrogels and boron complexes were cross-linked with and without glutaraldehyde, where the samples chemically cross-linked presented antibacterial and antifungal activities [37]. Electrospun PVA–chitosan–starch nanofibrous mats for wound dressings presented adequate mechanical properties and porosity to absorb aqueous fluids. They also presented antimicrobial properties against Gram-positive and Gram-negative bacteria [38].

Cellulose is a linear homopolymer obtained from the cell walls of plants and is insoluble in water. An acidic treatment can be performed on cellulose; when it is in contact with mono chloroacetic acid, the OH groups of cellulose are substituted by O–CH_2_COONa. The obtained product is sodium carboxymethyl cellulose (NaCMC), a polymer soluble in water [39]. PVA and NaCMC were blended and physically cross-linked and loaded with fucidic acid to serve as hydrogels for wound dressing. The addition of NaCMC led to a higher degree of swelling, elasticity, and porosity compared to PVA gels [40]. The 2.5% PVA, 1.125% NaCMC, and 0.2% sodium fucidate gel presented a high rate of healing in rats’ wounds [41]. Freeze–thawed PVA/polyethylene oxide/CMC gels loaded with tetracycline hydrochloride and natural agents (curcumin or *Aloe vera*) were porous and exhibited cumulative drug delivery, and they also presented antimicrobial properties [42]. 

Hydrogels that mimic the human skin could promote accelerated healing. A two-layered PVA hydrogel has similar mechanical properties to skin [43]; a three-layered sodium alginate–chitosan–Ca-polyglycolic acid hydrogel for Ca^2+^ release on the wound site showed 1000% water uptake and stimulated re-epithelization [44]. Laminated PVA hydrogels, in which each layer is submitted to different numbers of freeze–thawing cycles, is a potential drug delivery device able to sustain a constant delivery rate. Such gels were successfully loaded with Bovine Serum Albumin (BSA) protein, and its release rate was similar for the layer with three freeze–thawing cycles and for the layer with five freeze–thawing cycles [45]. Layered hydrogels can be loaded with natural product, such as honey, to stimulate the burn healing. The goal of the present work was to produce layered PVA–gelatin, PVA–starch, and PVA–CMC hydrogels loaded with manuka honey. These gels were characterized microstructurally using FTIR, and thermally using DSC; they were also evaluated for swelling capacity and biodegradation and hydrolysis. Morphology was assessed using SEM. A mathematical model was used to understand the behavior of the honey delivery. Antibacterial activity of the samples against *S. aureus* was also assessed.

## 2. Materials and Methods

### 2.1. Materials

The materials used were polyvinyl alcohol (PVA, molecular weight (Mw) 85,000–124,000 Da, 99% hydrolyzed, Sigma-Aldrich, Louis, MO, USA), gelatin (Sigma-Aldrich, Louis, MO, USA), sodium carboxymethyl cellulose (NaCMC, Mw ~250,000 Da, Sigma-Aldrich, Louis, MO, USA), potato starch (Sigma-Aldrich, Louis, MO, USA), and manuka honey (Manuka Doctor 20+, New Zealand).

### 2.2. Preparation of Samples

The samples were prepared in layers using a casting method. The polymers were dissolved at ~80 °C for 4 h under mechanical stirring (equipment Fisatom 710) (Fisatom Scientific Equipment Ltda., São Paulo, Brazil). The honey was added when the solutions reached room temperature, while stirring was maintained. This was followed by freeze–thawing cycles, where the first layer deposited was submitted to three cycles, the second layer (placed above the first layer) was submitted to two cycles, and the third layer (placed above the second layer) was submitted to one cycle. The composition and freeze–thaw protocol of the different samples is displayed in Table 1 and Table 2. The medium was varied for each layer to present a graduated concentration of honey, intended to generate a smoother release profile. The samples were dried in an oven (Fabbe Primar Industrial Ltda., São Paulo, Brazil) at 50 °C for 30 h.

### 2.3. Physicochemical Analysis

The physicochemical analysis of the samples was performed using Fourier-transform infrared spectroscopy (FTIR) with a Perkin-Elmer Spectrum 100 (Perkin-Elmer, Boston, USA) at COPPE/UFRJ in ATR mode, with a wavenumber range of 4000–600 cm^−1^, 32 scans/sample, and a spectral resolution of 4 cm^−1^. 

### 2.4. Thermal Analysis

The thermal analysis was conducted using a DSC Q200 (TA Instruments, New Castle, DE, USA) at EMBRAPA. For each analysis, ~10 mg of each sample was weighed. The heating rate used was 10 °C/min, from 40 °C to 230 °C. To overcome the thermal history of the samples (interference from the samples’ previous thermal processing), the second heating cycle was used to obtain the glass transition temperature (Tg), the melting temperature, and the degree of crystallinity (Xc), calculated according to Equation (1) [46]. The Xc calculated was based on relativity to the melting of PVA crystallites, where ΔH is the enthalpy of the sample peak at ~220 °C, w_t_ is the amount of PVA in the sample, and ΔH* is the theoretical enthalpy of 100% crystalline PVA (138.6 J/g) [47].

(1) $$X_{c}=\frac{100\;\Delta H}{w_{t}\Delta H^{*}}$$

### 2.5. Morphological Analysis

The fracture surfaces of the samples were morphologically evaluated using scanning electron microscopy (SEM) (TM3030Plus, Hitachi, Tokyo, Japan). The dried samples were sectioned when exposed to N_2_ (gaseous) atmosphere, and they were sputter-coated with silver (Ag^0^) (SCD005 Sputter Coater BAL-TEC, sample exposition for 150 s) and examined in a TM3030Plus Hitachi at CETEM. (Centre of Mineral Technology, Rio de Janeiro, São Paulo, Brazil) operating under high vacuum at 15 kV. The images were acquired in the backscattered electron mode (BSE).

### 2.6. In Vitro Analysis

The in vitro analysis involved swelling tests in saline solution. The honey release was evaluated using UV−Vis spectroscopy (Even, São Paulo, Brazil), and microbiological tests were conducted in the presence of *Staphylococcus aureus* bacteria. 

To perform the swelling tests, the samples were cut and weighed (where the samples weights were similar and triplicates were tested for each composition). They were immersed in a constant volume of sterile saline solution (10 mL) at room temperature and the samples were removed from the media at pre-determined intervals (0.5 h, 1 h, 2 h, 4 h, 24 h, 48 h, 72 h, and 96 h). The adsorbed water was dried using filter paper and the samples were weighed. The swelling degree (SD) was calculated according to the following equation:

(2) $$SD=100\left(W_{I}-W_{D}\right)/W_{D}$$

where W_I_ is the weight of the swollen sample and W_D_ is the weight of the initially dried sample [48,49]. After the four days of immersion, the samples were dried in an oven (50 °C, 24 h) and weighed. The gel fraction (GF), which measures the gel’s stability (the effectiveness of crystallites and chain entanglements in maintaining the gel’s structural integrity after immersion), was calculated according to Equation (3), where W_DS_ is the weight of the dried samples after the swelling test [49]. The sample’s weight loss (WL; the degradation of the samples due to the free chains carried to the media by the saline solution leaching) was defined by Equation (4) [48].

(3) $$GF=\frac{100w_{DS}}{w_{D}}\;\left(\%\right).\;$$

(4) $$WL=\frac{100\left(w_{D}-w_{DS}\right)}{w_{D}}$$

The microbiological test was performed following an adaptation of the standard ASTM E2180-07(2012) using *Staphylococcus aureus* (ATCC 6538). A suspension of *S. aureus* was prepared adjusting the turbidity to 0.5 on the MacFarland scale (correspondent to 10^8^ CFU/mL where CFU denotes colony forming unit). Next, 1 mL of the suspension was inoculated into 100 mL of the agar paste, resulting in 10^6^ CFU of *S. aureus* per mL of paste. The samples were placed in wells (24-well plate), where 200 μL of the referred agar paste was dropped in each well. The plate was incubated at 30 °C for 24 h. After incubation, the samples were transferred to Falcon tubes and decimal dilutions were prepared. Subsequently, the viability of the organism was evaluated by plating on PCA agar using the micro dropping technique and microcolonies were counted following incubation at 35 °C for 24 h.

The honey activity was estimated using the agar diffusion method. Various dilutions of honey (from 100% to 5%) were prepared. Their activity was determined in agar plates previously inoculated with 10^6^ CFU/mL. The following step was incubation at 37 °C for 24 h. The diameter of inhibition halos was measured.

### 2.7. Mathematical Model—Honey Delivery

To evaluate the honey release kinetics, the samples (of similar weight) were immersed in deionized water (10 mL) for two days. The media (aliquot of 3 mL) was removed in the same time intervals described in the swelling analysis. The aliquot was evaluated using quartz cuvettes in a spectrophotometer operating in the wavelength from the ultraviolet to visible light range (UV–Vis, equipment Even, wavelength of 500 nm) and the aliquot was returned to the sample media. To quantify the samples’ honey delivery, a calibration curve was plotted by reading the absorbance at 500 nm of aqueous solutions containing known amounts of honey, (0.05, 0.10, 0.15, 0.20, 0.25, 0.30, and 0.35% honey). To evaluate the honey delivery kinetics, the Korsmeyer–Peppas model was used (Equation 5, where M_t_/M_inf_ is the honey concentration release at time *t*, *k* is the release rate (constant), and *n* is the release exponent) [50], while the coefficient of diffusion (D) was evaluated using a modified Fick’s law equation (Equation (6), where δ is the sample’s thickness/2) [51]. The mean standard deviation (DRM) was calculated according to Equation (7), where exp is the experimental value while calc is the value estimated.

(5) $$\frac{M_{t}}{M_{inf}}=kt^{n}.$$

(6) $$\frac{M_{t}}{M_{inf}}=\sqrt{\frac{2Dt}{\pi \delta }}.$$

(7) $$DRM=\frac{\sum^{\;}\frac{\left|exp-calc\right|}{exp}}{n}$$

## 3. Results and Discussion

### 3.1. Physicochemical Analysis

**Figure 1:**
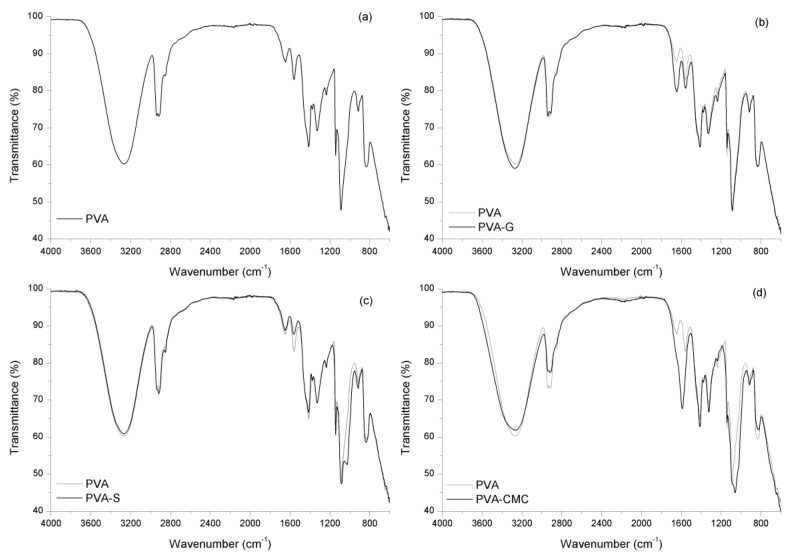
Spectra of PVA (polyvinyl alcohol) blends: (**a**) PVA, (**b**) PVA-G (poly vinyl alcohol – gelatin), (**c**) PVA-S (poly vinyl alcohol –starch) and (**d**) PVA-CMC (polyvinyl alcohol – sodium carboxymethyl cellulose).

Physicochemical analysis of the samples through FTIR was intended to reveal the interactions between the polymers themselves and between polymers and honey. The PVA sample exhibited its characteristic bands (Table 3, Table 4, and Figure 1). The main differences between the spectra for PVA–gelatin (PVA-G) and PVA only would be that the PVA-G spectrum presents considerably more intense bands at 1646 cm^−1^ and at 1558 cm^−1^. These intense bands would represent the contribution of the vibrations of PVA groups, and also those of gelatin, where the band at 1646 cm^−1^ would be related to stretching C=O of amide I and the band at 1558 cm^−1^ would be due to bending –NH of amide II [52,53]. The PVA-G sample does not present a band at 1685 cm^−1^, indicating the esterification of gelatin’s carboxylic groups [54]. All samples’ FTIR bands are displayed in Table 4 and the band modes are discussed in the text.

The PVA–starch gel (Figure 1) exhibits bands that are more intense compared to the PVA sample. These bands could be related not only to PVA as previously described, but also to starch, e.g., the band at 2918 cm^−1^, a contribution of starch stretching of C–H bonding [63] and deformation of the CH_2_ bonding vibration [64] (which is a band at 2852 cm^−1^, indicative of the presence of protein in starch [65]). There are bands that are less intense than in the PVA sample, e.g., bands at 1560 and 1416 cm^−1^, the latter being the starch’s vibration of CH_2_ group [63]. The lower absorbance in this region could represent the physical interaction between PVA and starch, which may have diminished these bonds’ IR vibrations. In addition, there are bands related to starch only; bands at 1652 cm^−1^ are related to the vibration of H_2_O molecules adsorbed in the starch amorphous phase; stretching of C=O group related to amide I [64] at 1040 cm^−1^ could be the shift of the band at 1048 cm^−1^, related to starch crystalline phase [64]. The displacement of the band to lower wavenumbers could represent physical interaction, since it is related to the ratio of hydrogen bonding in the blend [63]. There is also a band at 1028 cm^−1^ due to starch amorphous phase [64]. 

PVA-CMC samples present bands that are more intense than the corresponding bands in the PVA sample (Figure 1), e.g., the bands at 1416 and 1324 cm^−1^, related to NaCMC’s –CH_2_ scissoring vibration and OH bending vibration, respectively [66]. There are bands related to NaCMC only, e.g., 1590 cm^−1^, regarding carbonyl group vibration [67], or it could even be the result of the COO^−^ group, whose bands would be at [66,68] 1604–1620 cm^−1^; there is also a band at 820 cm^−1^, due to NaCMC’s stretching C–O [69]. In the PVA-CMC sample, some bands are less intense than those in the PVA sample, e.g., the bands at 2936 cm^−1^ (NaCMC’s methylene’s CH_2_ stretching [70]), 2917 cm^−1^ (NaCMC’s C–H stretching [68]), and 830 cm^−1^. The last one could be the band related to NaCMC’s β-glucosidic group shifted to a lower wavenumber, indicating hydrogen bonding between PVA and NaCMC and miscibility of the polymers [66]. In addition, the band at 1052 cm^−1^ of NaCMC (asymmetric stretching of ether groups [70]) is displaced to 1060 cm^−1^ in the PVA-CMC sample. 

**Figure 2:**
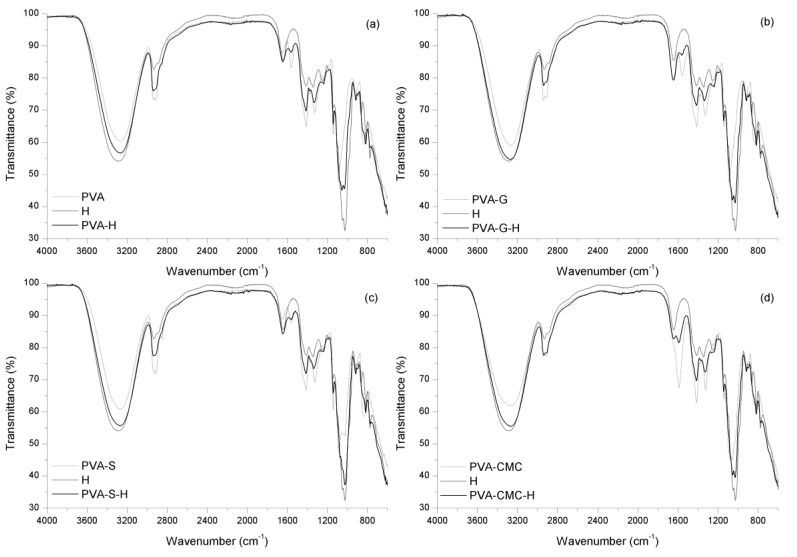
Spectra of PVA blends loaded with honey: (**a**) PVA, honey, and PVA incorporating honey; (**b**) PVA–gelatin, honey, and PVA–gelatin incorporating honey; (**c**) PVA–starch, honey, and PVA–starch incorporating honey; (**d**) PVA–CMC, honey. and PVA–CMC incorporating honey.

The PVA–honey sample presented bands which could be related to PVA as previously described, as well as to honey (Figure 2). Some bands present intermediary intensity (between the PVA band and that of honey); e.g., there are honey bands between 3270 cm^−1^ and 2921 cm^−1^, related to OH stretching of free water and to C–H stretching of sugars [53]; there is a band at 3270 cm^−1^, where honey’s primary amide N–H stretching could contribute to this band’s formation [71]; there is a band at 2940 cm^−1^, due to saccharide CH_2_ symmetric mode [72]. The sample also presents an intense band at 1645 cm^−1^, a region of carboxyl stretching of proteins [71]. The sample also presents bands at 1334 cm^−1^ and at 1250 cm^−1^, related to C–N stretching [71] and to C–C–H deformation [72]. The sample’s bands in the region of 1090–700 cm^−1^ presents the PVA band at 917 cm^−1^, while the PVA band at 1090 cm^−1^ is shifted to 1076 cm^−1^ (honey’s C–O stretching [72]); the sample also presents the honey bands at 1055, 1031, 819, and 775 cm^−1^, due to C–O stretching [72], C–O and C–C stretching of sugars [53], saccharide C–O and C–C stretching (900–750 cm^−1^) [53], and C–H deformation [72], respectively.

The PVA-G-H sample presented some differences compared to the PVA-G spectrum, especially from 1560 cm^−1^ to lower wavenumbers (Figure 2). There are some bands in the PVA-G-H sample that are clearly related to the presence of honey, although most of them are slightly shifted, e.g., the bands at 1337, 1256, 1055, 1030, 895, 818, and 776 cm^−1^, as previously described. Nevertheless, there are some bands absent in the PVA-G-H spectrum: the band at 1378 cm^−1^, related to PVA’s wagging –CH_2_– [60] and the band at 834 cm^−1^, due to PVA chain stretching C–C [56]. In addition, there is a band at 1337 cm^−1^, which could be the result of the overlapping/displacement of the band at 1328 cm^−1^ related to PVA-G, and the band at 1343 cm^−1^ related to honey. The band at 1551 cm^−1^ is present only in the PVA-G-H sample (it is not related to PVA-G or to honey). Nevertheless, gelatin usually presents a band at 1550 cm^−1^ related to amide II vibration [73], where the presence of honey could have altered the material’s interactions allowing this band’s vibration in PVA-G-S sample. The band at ~1100–1090 cm^−1^, related to both honey and PVA-G, is shifted to 1073 cm^−1^ as a shoulder in the PVA-G-H sample. The shift in the position of the bands and the altering of intensity could represent a physical interaction between components. The altering of the original sample bands with the addition of drugs can be exemplified by the addition of salicylic Acid to PVA-G, where some bands are absent after this addition [54].

The PVA-S-H sample presented similar bands to those of the PVA-S sample, although some bands are near the honey band positions (Figure 2). The overlap between the PVA-S and honey bands could result in the bands’ displacement, e.g., the band at 2918 cm^−1^ of the PVA-S sample (C–H bonding [63]) is shifted to 2922 cm^−1^ in the PVA-S-H sample (there is a honey band at 2929 cm^−1^, C–H stretching of sugars/carbohydrates [53,74]). There are some bands related only to honey, and some of them present a slight displacement, e.g., at 1337, 1259, and 1026 cm^−1^. The honey band at 1098 cm^−1^ and the PVA-S band at 1084 cm^−1^ were shifted toward 1074 cm^−1^ in the PVA-S-H sample, where this band displacement could represent the interference/interaction of honey with the PVA-S blend, results similar to those found when citric acid was added to a PVA-S sample [75]. There is a band at 1551 cm^−1^ in the PVA-S-H sample, which is not apparent in either PVA-S or honey analysis, suggesting a probable chemical interaction. 

In the PVA-CMC-H sample (Figure 2), there are bands related to PVA-CMC and bands related to honey (e.g., bands at 1644, 1261, 1030, 899, and 775 cm^−1^). The PVA-CMC band at 1378 cm^−1^ (wagging of –CH_2_– [60]) was shifted to a lower wavenumber in the PVA-CMC-H sample, 1373 cm^−1^. In addition, there were bands of PVA-CMC and honey that could be overlapped in the PVA-CMC-H sample, e.g., the band at 3271 cm^−1^ in the PVA-CMC-H sample would be a contribution of the PVA-CMC band at 3264 cm^−1^ and of the honey band at 3289 cm^−1^. Bands related to the loaded material, as well as to PVA and carboxymethyl cellulose, show that the added material was properly loaded [76]. Nonetheless, the bands at ~2929–2917 cm^−1^, as a contribution of PVA-CMC and honey, were a lower wavenumber, 2911 cm^−1^, which could represent the physical interaction. 

In summary, in all samples, the presence of all the intended components was observed. In addition, the PVA-G sample exhibited esterification of gelatin’s carboxylic groups; in the PVA-S analysis, physical interaction was observed between polymers; PVA-CMC samples exhibited hydrogen bonding between polymers; PVA-G-H samples presented physical and probably chemical interactions between components, while PVA-S-H and PVA-CMC-H exhibited only physical interaction between materials.

### 3.2. In Vitro Analysis—Swelling Analysis

The samples were immersed in saline solution for four days. The samples were stretched by the media’s ingress, followed by a relaxation of the polymer chains [77]. When the samples reached a plateau (at 48 h), the elastic and osmotic forces are balanced, a state known as the equilibrium of swelling degree (ESD) [78]. PVA-CMC samples swelled more than any other sample (*p* < 0.05). The PVA-S samples presented the lowest swelling among the samples without honey (*p* < 0.05). PVA samples swelled significantly more than PVA-H samples (*p* < 0.05). PVA-S-H and PVA-H swelled significantly less than the PVA-CMC-H and PVA-G-H samples (*p* < 0.05). The presence of honey diminishing the PVA gel’s ability to swell could be caused by honey occupying the hydrogel pores, limiting the space available to be filled by fluid when the gels were immersed in saline solution [70]. NaCMC presents high fluid uptake [79], which could enhance a gel’s ability to swell. In gelatin–starch films, gelatin decreased the starch films’ moisture content due to the affinity between the polymers and due to the formation of hydrogen bonding between gelatin and starch [80], which indicates a low interaction between PVA and gelatin. Starch can be considered a highly hydrophilic material, which would increase the PVA hydrogel’s swelling capability [81], although gelatin presents a higher impact on the PVA gel swelling than starch [82].

**Figure 3:**
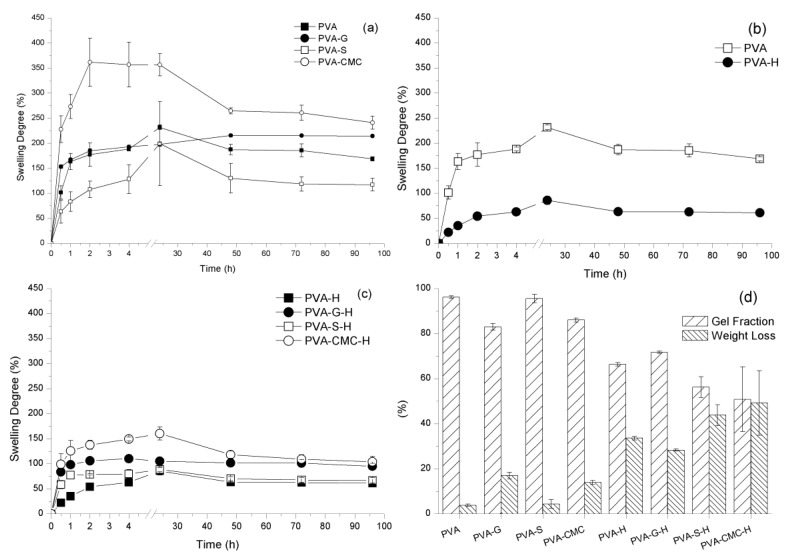
Sample swelling degree (**a**–**c**); sample degradation and gel fraction (**d**).

Weight loss was significantly higher for samples containing honey than for samples without honey (*p* < 0.05). This trend could indicate some degradation of the biomaterial, but could also be due to weight loss due to honey delivery [83]. The samples without honey presented similar weight loss (*p* > 0.05) to each other, probably related to the cleavage of chain entanglements [84]. PVA-CMC-H presented higher weight loss than PVA-G-H (*p* < 0.05). In addition, material was delivered to the media regardless of the presence or absence of honey. The samples presented hydrolytic degradation in media, since the PVA blends are hydrophilic and the aqueous media can break the chains entanglements. This could also interfere with the cross-linking points/crystallites, which leads to degradation and chain leach out by the media [85]. The samples incorporating honey seem to have experienced both honey delivery and degradation (Figure 3). Gel fractions followed the opposite trend of weight loss, where the highlight would be that samples without honey presented higher gel fractions than samples with honey. Honey could be located physically between the polymer chains diminishing the contact between them, causing the degree of crystallinity/cross-linking, as well as entanglements, to be diminished [84,86]. 

### 3.3. Mathematical Model—Honey Delivery

To evaluate the kinetics of honey delivery, the release of the samples with honey was analyzed after compensating for the release of materials from the samples without honey (the degradation of the matrices was measured to guarantee that only the delivery of honey was modeled). The samples presented the data shown in Table 5. The *R^2^* is the coefficient of determination and PVA-H had the highest value and the only one presenting only 60% of delivery (suitable to be modeled by Korsmeyer–Peppas equation [50]), although none of the samples’ compositions reached *R^2^* ≥ 0.97 [87]. All the samples presented *n* < 0.5 probably indicating a Fickian diffusion, where the Fickian diffusion is characterized by *n* = 0.5 and anomalous non-Fickian transport is characterized by 0.5 < *n* < 1.0. According to the *R^2^* and the *n* values, the PVA-H hydrogel presented the best fit, although the high mean standard deviation (DRM) of the analysis probably interferes with the honey delivery modeling [88]. The *D* value was high for PVA-S-H, which presented a relatively low GF among the samples with honey, indicating that the samples presenting low cross-linking/entanglements probably present bigger pores, which facilitates the honey diffusion [89]. The highest *D* was exhibited by PVA-S-H samples, while the lowest *D* was the one related to PVA-H samples. The PVA-S-H samples were amorphous gels, while the PVA-H presented some crystallinity. Amorphous gels would present higher network stretching with the media entrance than semi-crystalline ones, facilitating the honey diffusion.

In summary, the samples reached equilibrium swelling in 48 h, while the PVA-CMC sample presented the highest SD, probably due to NaCMC’s highly hydrophilic character. PVA-(CMC or G)-H presented a higher swelling capacity than PVA-H and PVA-S-H. Samples with honey also presented a lower gel fraction than samples without it. The addition of honey might have stimulated a physical interaction between components, but its presence between chains could also diminish the chain interaction between polymers (lower entanglement and/or crystallites). The samples loaded with honey not only presented low GF, but the low GF also resulted in a high diffusion coefficient, indicating that the amount of crystallites/entanglements interferes with the honey delivery kinetics.

### 3.4. Thermal Analysis

**Figure 4:**
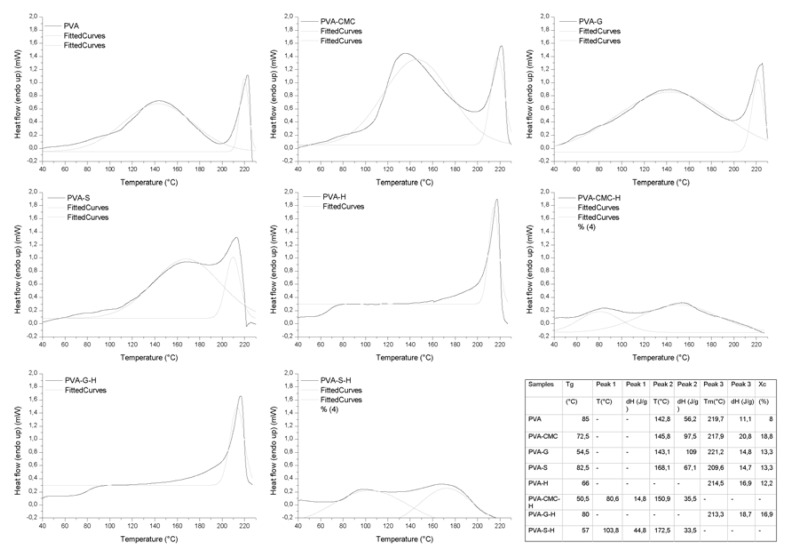
DSC curves of the samples.

The transition temperatures (glass transition temperature (Tg), melting temperature (Tm)), enthalpies (ΔH), and degree of crystallinity (Xc) are displayed in Table 6 and Figure 4. Regarding the samples without honey, the gels presented Tg and two endothermic peaks (peaks 2 and 3). The first peak could be attributed to water evaporation (free and bonded H_2_O), while the second peak could be attributed to the melting of PVA crystallites [47,90]. In all samples, the energy (ΔH) required for evaporation increased with the addition of the natural polymers, especially for PVA-S, which presented a high GF. The added polymers presented additional groups, along with the PVA groups, that could be solvated by water molecules. The natural polymers presented hydrophilic characteristics. The H_2_O molecules could be strongly attached to the polymers, where high energy is required for evaporation.

The addition of NaCMC slightly diminished the Tg and Tm of the samples. The interaction between NaCMC chains and PVA chains diminished the ability of PVA chains to pack without structural defects, diminishing Tm [91]. The lowering of the Tg would indicate non-compatibility between PVA and NaCMC and weak physical interaction between chains [92], confirmed by FTIR. The sample having a lower Tg is consistent with the GF results, where low entanglement leads to low GF. The Xc was higher than that of PVA and, since there is an indication of low interaction between chains, the PVA chains could be packed together in many crystallites presenting structural defects. 

The addition of gelatin to PVA led to higher Tm and Xc, as well as the presence of two glass transition temperatures. The first Tg (54.5 °C) could be attributed to gelatin, where this low Tg could be related to the presence of water molecules between the gelatin chains, and the second (82.5 °C) could be attributed to PVA [93]. The peak at ~143 °C could be attributed to evaporation, as previously discussed, as well as to the gelatin sol–gel transition, while the Xc of PVA in the PVA-G sample was high. The existence of the two glass transition temperatures, i.e., the possible peak related to gelatin and the second peak related to PVA melting temperature, highlights a probable phase separation between PVA and gelatin [94].

The addition of starch to PVA increased Tg and Xc but lowered the Tm. PVA and starch presented a physical interaction, according to the FTIR, as well as high GF and low WL, indicating that PVA and starch interacting contributed to the formation of crystals and to the entanglement of chains. A lowering in Xc and Tm was expected, since PVA and starch can interact and diminish the possibility of the organization of PVA chains, and the interaction would increase the sample’s Tg, since the amorphous chains, connected to each other, would have lower freedom of movement [95]. Nonetheless, the opposite effect of an increase of Tm and Xc with the addition of starch to PVA would represent an interaction and a synergic effect between the components [96]. 

The addition of honey to the PVA gel led to diminished Tg and Tm, although the Xc increased. The presence of honey and PVA led to the formation of more crystals, probably crystals presenting more structural defects than the PVA ones [97]. The diminished Tg would represent more freedom of movement for the amorphous chains. The presence of honey would physically hamper the formation of amorphous entanglements, diminishing the GF. The FTIR does not show interactions between components. 

PVA-G-H presented the PVA crystallinity peak, which could be attributable to phase separation between PVA and gelatin/honey [94], which could then stimulate contact and interactions between PVA chains, increasing not only Xc, but also GF. The weight loss was lowest among the samples with honey due to high Xc and GF. PVA-CMC-H and PVA-S-H did not present PVA crystallinity peaks, but they presented the water evaporation peak at high temperatures, probably due to the strong interaction between H_2_O molecules and the natural polymers and honey. They also presented peaks within a lower temperature range 80–105 °C, probably also attributable to evaporation. These samples presented low GF, no Xc, and high WL, indicating that the addition of honey interferes with polymer interactions, and especially the organization of PVA chains [70]. In addition, the gels’ structural integrity was maintained by the chains’ entanglement, since they presented low GF and the absence of crystallinity.

In summary, all the samples without honey presented PVA crystallization and the peak related to water evaporation. The gelatin presented phase separation when mixed with PVA (in the presence or absence of honey), where PVA had the ability to crystallize. PVA–starch presented physical interactions, while PVA-CMC probably presented low miscibility; however, PVA was able to crystallize in both. The addition of honey to PVA led to the presence of many PVA crystals with defects (indicated by a lower Tm when compared to the pure PVA sample). PVA-S-H and PVA-CMC-H were amorphous gels. 

### 3.5. Morphological Analysis

**Figure 5:**
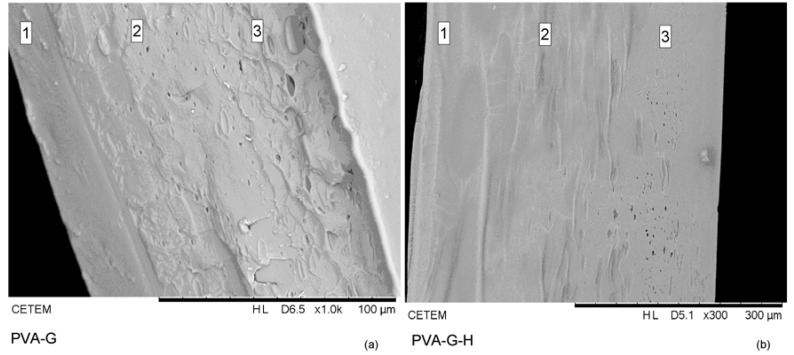
SEM of (**a**) PVA-G and (**b**) PVA-G-H samples.

The SEM analysis, represented by the PVA-G and PVA-G-H images (Figure 5), showed some differences over the sectioned surface, which probably indicate the different layers in the samples without honey. Although there were no distinguishable interfaces between layers, there was adhesion between layers [98]. The first layer (“1”, three freeze–thawing cycles) would be less porous (probably the densest part of the gel), followed by the second layer (“2”, two freeze–thawing cycles) and the third layer (“3”, one freeze–thawing cycle), the most porous part of these gels. The increase in number of freeze–thawing cycles (and associated physical cross-linking) leads to less porosity and higher density [99,100]. In the samples with honey, the different layers were difficult to distinguish, probably due to honey being located in the sample pores. The densest layer would be the external layer of the gel, where there is a probable low diffusivity of honey, while the second layer would the honey source; in the third layer (porous), honey could diffuse to the wound [98].

### 3.6. In Vitro Analysis—Microbiological Analysis

**Figure 6:**
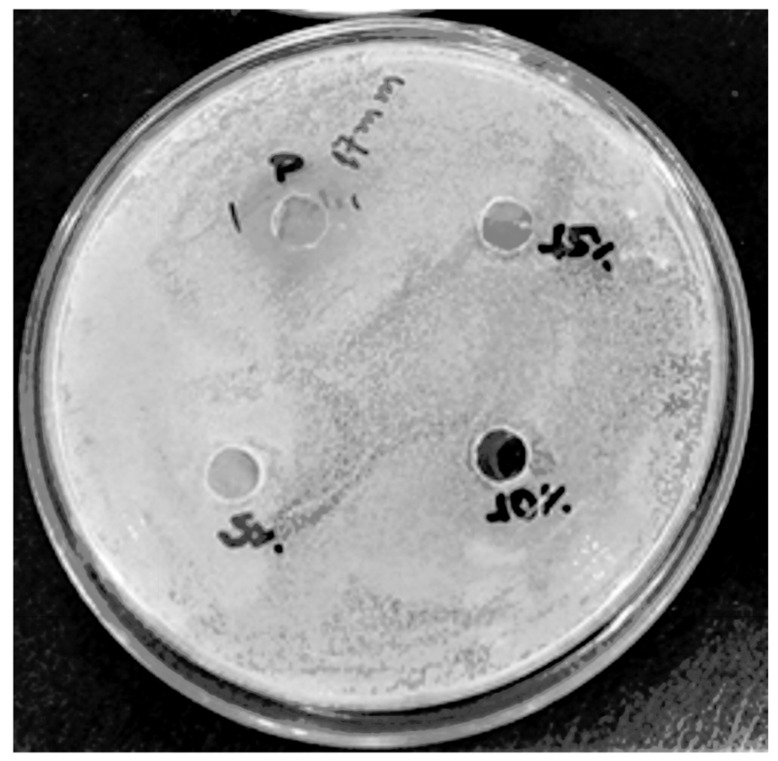
Halo of inhibition of manuka honey against *Staphylococcus aureus* (P = pure honey, showing 17-mm diameter of inhibition).

**Figure 7:**
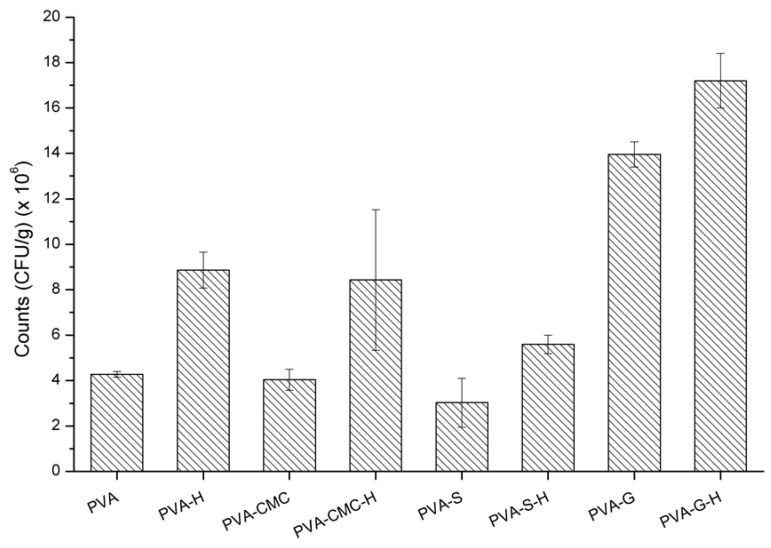
Antimicrobial analysis regarding the samples’ activity.

The bacterial growth in the presence of all samples containing honey was compared to the respective samples without it (Table 7). The incorporation of honey into the polymers was ineffective against *S. aureus*, since the reduction in the amount of *S. aureus* was negligible (less than one log cycle). None of the samples were bactericidal or even bacteriostatic. PVA polymer is biocompatible [101], gelatin is bioresorbable [102], NaCMC is bioactive [103], and starch is also biocompatible [104]; consequently, they are inert and have no biocidal action. In order to determine if the amount of honey was the limiting factor for the bactericidal effect, honey dilutions were prepared and tested against *S. aureus.* Pure manuka honey presented activity against these microorganisms (it was bactericidal) and dilutions superior to 25% honey were bacteriostatic (Table 7 and Figure 6 and Figure 7). More diluted solutions did not present activity against *S. aureus*, which indicates that the amount of honey used in the samples was not enough to inhibit these organisms. Based on the amount of sugar that presents activity against *S. aureus* (bacteriostatic or bactericidal), concentrations superior to 22% honey are required, although diluted solutions of manuka honey (~3% manuka) can be bactericidal [105]. Although high concentrations of manuka honey were required for activity in the present work, other authors found out that concentrations of 6% (*w*/*v*) manuka honey could be considered the minimum inhibitory concentration of manuka honey [106]. In the present work, it was observed that 25% manuka honey solutions usually presented activity against *S. aureus*, probably due to the non-peroxide components [105]. Manuka honey is also usually used as a co-adjuvant to antibiotics in treatment against *S. aureus* [107], although the effectiveness of honey is dependent on its concentration [11].

## 4. Conclusions

Layered cryogels based on polyvinyl alcohol and starch (PVA-S), gelatin (PVA-G), and sodium carboxymethylcellulose (PVA-CMC) were fabricated and subjected to detailed characterization with respect to chemical and physical interactions and also their abilities to act as a matrix for honey release in an aqueous media. Esterification of gelatin’s carboxylic groups was observed in the PVA-G sample, whereas other samples exhibited a variety of physical interactions and, in the case of PVA-G-H (honey laden), some probable chemical interactions.

PVA-CMC presented the highest swelling degree (SD) of all, probably due to NaCMC’s highly hydrophilic nature. PVA-CMC-H and PVA-G-H presented the highest swelling capacities of the honey laden samples, which has relevance for the envisaged wound-care applications.

The cryogels with honey presented lower gel fractions than unladen ones. It is hypothesized that the presence of honey could hinder interactions among the constituent polymers, causing a lower degree of entanglement and/or fewer crystallites, thereby also increasing the gels’ coefficients of diffusion with respect to honey and other substances. 

Gelatin exhibited phase separation when mixed with PVA in the presence or absence of honey, but the PVA still had the ability to crystallize. PVA–starch presented physical interactions, while PVA-CMC probably presented low miscibility, but the PVA was able to crystallize in both. The addition of honey to PVA led to the presence of many PVA crystals with defects (lower Tm when compared to PVA sample). PVA-S-H and PVA-CMC-H were amorphous gels. In addition, the interfaces between the layers of the samples were not readily distinguishable in SEM images, indicating continuity between layers, which helps the maintenance of the gel structural integrity. This is quite likely due to the formation of physical entanglements and hydrogen bonds at the interface during freeze–thawing, and may also include the formation of new PVA crystals to act as networking points and potentially form new cross-links across the interface.

Cryogel samples with or without honey were not found to be active against *S. aureus*. The honey itself was bactericidal, while honey solutions presenting more than 25% honey were bacteriostatic. The final conclusion is that gels loaded with at least 25% manuka honey may be required for the application, and that cryogels such as those in this paper have the potential to act as the delivery systems, with further development to allow higher loadings without compromising structural integrity.

## Figures and Tables

**Table 1:** Layer-wise composition of media (honey and water) and freeze–thaw cycle parameters.

Layers	Medium		Freeze–Thawing Cycles
	Honey (mL)	Water (mL)	
Samples without honey			
First	0	100	3× (1 h at −16 °C and 40 min at room temperature)
Second	0	100	2× (1 h at −16 °C and 40 min at room temperature)
Third	0	100	1× (1 h at −16 °C and 40 min at room temperature)
Samples with honey			
First	0	100	3× (1 h at −16 °C and 40 min at room temperature)
Second	10	90	2× (1 h at −16 °C and 40 min at room temperature)
Third	5	95	1× (1 h at −16 °C and 40 min at room temperature)

**Table 2:** Composition of samples according to polymer content.

Samples	PVA (g)	NaCMC (g)	Gelatin (g)	Starch (g)	Medium (mL)
PVA	10	-	-	-	100
PVA-CMC	8	2	-	-	100
PVA-G	8	-	2	-	100
PVA-S	8	-	-	2	100
PVA-H	10	-	-	-	100
PVA-CMC-H	8	2	-	-	100
PVA-G-H	8	-	2	-	100
PVA-S-H	8	-	-	2	100

**Table 3:** PVA’s Fourier-transform infrared spectroscopy (FTIR) bands and vibration modes.

Bands (cm^−1^)	PVA Group Vibration Mode
3272	stretching –OH of hydrogen bonded hydroxyl groups [55]
2940	stretching C–H [56]
2919	antisymmetric stretching of C–H from alkyl groups [57]
2852	symmetric stretching of C–H from alkyl groups [57]
1646	bending HOH [58]
1559	stretching C=C [59]
1415	bending CH_2_ [56]
1378	wagging of –CH_2_– [60]
1328	bending –C–H– and –O–H– [60]
1237	stretching C–C [61]
1143	PVA crystallite formation [55] and stretching C–C and C–O–C [56]
1090	Out-of-plane C–O vibration [62]
916	rocking of CH_2_ vibration [56]
834	stretching C–C [56]

**Table 4:** Samples FTIR bands, where “G” is gelatin, “S” is starch, “CMC” is sodium carboxymethyl cellulose, and “H” is manuka honey.

PVA	PVA-G	G	PVA-S	S	PVA-CMC	CMC	PVA-H	PVA-G-H	PVA-S-H	PVA-CMC-H
(cm^−1^)										
3272	3272	3284	3272	3292	3264	3310	3270	3271	3273	3271
		3078								
2940	2940	2944	2937	2929	2936		2940	2939	2937	2939
2919	2911		2918		2917	2920	2921	2913	2922	2911
2852		2877	2851			2886				
			1652				1651			
1646	1646	1626	1646	1635			1645	1643	1643	1644
					1590	1586				1591
1559	1558	1535	1560				1560	1560	1560	
		1450						1551	1551	
1415	1412	1401	1416	1421	1416	1413	1416	1416	1416	1416
1378	1378		1377		1378		1376			1373
		1334		1338		1321	1334	1337	1337	
1328	1328		1328		1324					1326
						1268	1264	1256	1259	1261
1237	1238	1239	1237		1238		1238	1240	1239	1241
		1202				1205				
1143	1142	1161	1143	1152	1142		1143	1143	1144	1143
				1124		1100				
1090	1088	1082	1084	1078	1086		1076	1073(s)	1074	1075(s)
		1030	1040	1045	1060	1052	1055	1055	1048(s)	1054
		972	1028	1005		1021	1031	1030	1026	1030
916	916	921	917	926	915		917	918	918	917
						895		895	897	899
834	834		836		829					
					820		819	818	818	818
							775	776	771	775

**Table 5:** Kinetics parameters of the model representing honey delivery from the samples.

Samples	M_t_/M_inf_ (%)	*n*	*K* (%/min)	*R^2^*	DRM (%)	*D* (× 10^−2^ mm^2^/min)
PVA-H	60	0.26	15	0.95	17.65	0.0201
PVA-S-H	88	0.076	60	0.84	6.96	0.0487
PVA-CMC-H	83	0.1	50	0.90	6.7	0.0343
PVA-G-H	95	0.03	80	0.87	2.5	0.0350

**Table 6:** DSC data of the evaluated samples.

Sample	ESD	GF	WL	Tg (°C)	Peak 1		Peak 2		Peak 3		
	(%)				T (°C)	ΔH (J/g)	T (°C)	ΔH (J/g)	Tm (°C)	ΔH (J/g)	Xc (%)
PVA	187.3 ± 10.3	96.1 ± 0.5	3.8 ± 0.5	85	-	-	142.8	56.2	219.7	11.1	8.0
PVA-CMC	264.6 ± 6.0	86.0 ± 0.8	13.9 ± 0.8	72.5	-	-	145.8	97.5	217.9	20.8	18.8
PVA-G	215.4 ± 2.6	82.9 ± 1.4	17.0 ± 1.4	54.5	-	-	143.1	109.0	221.2	14.8	13.3
PVA-S	130.1 ± 29.4	95.5 ± 1.8	4.4 ± 1.8	82.5	-	-	168.1	67.1	209.6	14.7	13.3
PVA-H	63.4 ± 1.0	66.3 ± 0.8	33.6 ± 0.8	66	-	-	-	-	214.5	16.9	12.2
PVA-CMC-H	118.4 ± 7.2	50.8 ± 14.3	49.1 ± 14.3	50.5	80.6	14.8	150.9	35.5	-	-	-
PVA-G-H	101.9 ± 7.2	71.7 ± 0.4	28.2 ± 0.4	80	-	-	-	-	213.3	18.7	16.9
PVA-S-H	70.2 ± 3.9	56.2 ± 4.6	43.7 ± 4.6	57	103.8	44.8	172.5	33.5	-	-	-

**Table 7:** Antimicrobial analysis regarding the samples and manuka honey dilutions.

Samples	Counts (CFU/g) (× 10^6^)
PVA	4.28 ± 0.13
PVA-H	8.86 ± 0.79
PVA-CMC	4.04 ± 0.46
PVA-CMC-H	8.43 ± 3.10
PVA-S	3.03 ± 1.08
PVA-S-H	5.59 ± 0.41
PVA-G	13.95 ± 0.55
PVA-G-H	17.20 ± 1.20
Manuka Honey dilution	
(Honey/water)	Halo (diameter)
100% (pure H)	(17 ± 1.4) mm
35–25%	Bacteriostatic
20–5%	No activity
